# Human Wagering Behavior Depends on Opponents' Faces

**DOI:** 10.1371/journal.pone.0011663

**Published:** 2010-07-21

**Authors:** Erik J. Schlicht, Shinsuke Shimojo, Colin F. Camerer, Peter Battaglia, Ken Nakayama

**Affiliations:** 1 Department of Psychology, Harvard University, Cambridge, Massachusetts, United States of America; 2 Department of Biology, California Institute of Technology, Pasadena, California, United States of America; 3 California Institute of Technology, Department of Economics, Pasadena, California, United States of America; 4 Massachusetts Institute of Technology, Department of Brain and Cognitive Sciences, Cambridge, Massachusetts, United States of America; University of Minnesota, United States of America

## Abstract

Research in competitive games has exclusively focused on how opponent models are developed through previous outcomes and how peoples' decisions relate to normative predictions. Little is known about how rapid impressions of opponents operate and influence behavior in competitive economic situations, although such subjective impressions have been shown to influence cooperative decision-making. This study investigates whether an opponent's face influences players' wagering decisions in a zero-sum game with hidden information. Participants made risky choices in a simplified poker task while being presented opponents whose faces differentially correlated with subjective impressions of trust. Surprisingly, we find that threatening face information has little influence on wagering behavior, but faces relaying positive emotional characteristics impact peoples' decisions. Thus, people took significantly longer and made more mistakes against emotionally positive opponents. Differences in reaction times and percent correct were greatest around the optimal decision boundary, indicating that face information is predominantly used when making decisions during medium-value gambles. Mistakes against emotionally positive opponents resulted from increased folding rates, suggesting that participants may have believed that these opponents were betting with hands of greater value than other opponents. According to these results, the best “poker face” for bluffing may not be a neutral face, but rather a face that contains emotional correlates of trustworthiness. Moreover, it suggests that rapid impressions of an opponent play an important role in competitive games, especially when people have little or no experience with an opponent.

## Introduction

**Figure 1:**
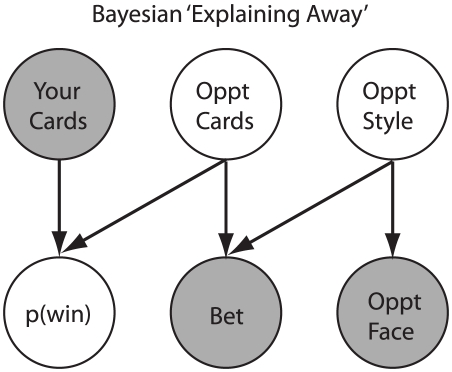
Diagram shows a Bayesian network for the poker task used in this experiment. White nodes are hidden variables, and gray nodes correspond to observable information. Arrows between nodes represent conditional relationships between the variables. In this scenario, the probability of winning the bet amount is based on the subject's starting hand (observable variable) and their opponent's hand (hidden variable). Since subjects cannot directly observe their opponent's hand, they can use the fact that the opponent bet (observable variable) to put them on a ‘range’ of possible hands. However, in order to do this accurately, they must have an estimate of their opponent's style of play (hidden variable). More specifically, the probability of a particular hand winning is lower against an opponent who only bets with high-value hands, compared to an opponent who frequently bluffs (i.e, bets with poor hands). Since the opponent's style is also a hidden variable, subjects can use an opponent's face information (observable variable) to estimate their style. This process is called Bayesian ‘explaining away’, as the opponent's face explains-away the possibility of a bluff being the cause of an opponent's bet.

When participating in a competitive wagering with an unfamiliar individual, rapid impressions of the opponent are formed through observable information, and depending on the situation, different attributes become important to estimate. For example, success in a poker game is limited by a player's ability to estimate their opponent's strategy. Since an opponent's strategy cannot be directly observed, it must be inferred through auxiliary information (e.g., facial properties). This inferential process is deeply related to concepts in Bayesian explaining away (e.g., [1], [2]), which provides a formal framework for how information is used to arrive at estimates of hidden variables (Figure 1). Theory and experimentation in cooperative games has demonstrated that people change their wagering behavior according to rapid evaluations of a partner, even when there has been no previous history with the individual [3], [4]. Such automatic impressions are thought to be processed by areas of the brain that are involved with emotional decision making [5]–[8].

Once rapid impressions have been formed, beliefs can later be updated by direct experience with the individual, to develop a new estimate that will be used going forward. Within the poker scenario provided above, experience could include return on investment percentages achieved against a particular opponent. In fact, research in strategic games has explored how wagering decisions are modified through experience with a partner. In repeated trust games, people's willingness to share money with a partner is strongly influenced by both previous return rates [9]–[12] and the probability of wagering with the same partner in future negations [13]. It is also known that areas of the brain responsible for people's experienced-based impressions of a partner are the same areas that are known to be involved in predicting future reward [14]–[16], and that these regions activate differently in autistic adults [17].

In all of these studies, subjective estimates of a partner are used to modify wagering decisions in an economic situation that is mutually beneficial to both parties. However, little is known about how rapid impressions of an opponent, based on face information, operate and influence behavior in competitive (i.e., zero-sum) games, where one person's gain is another person's loss. In fact, research and theory in competitive games has exclusively focused on how opponent models are developed through previous outcomes (i.e., the likelihood), and how peoples' decisions relate to normative predictions [18]–[25]. This study is the first to explore if rapid estimates of opponents are used in competitive games with hidden information, even when no feedback about outcomes is provided.

**Figure 2:**
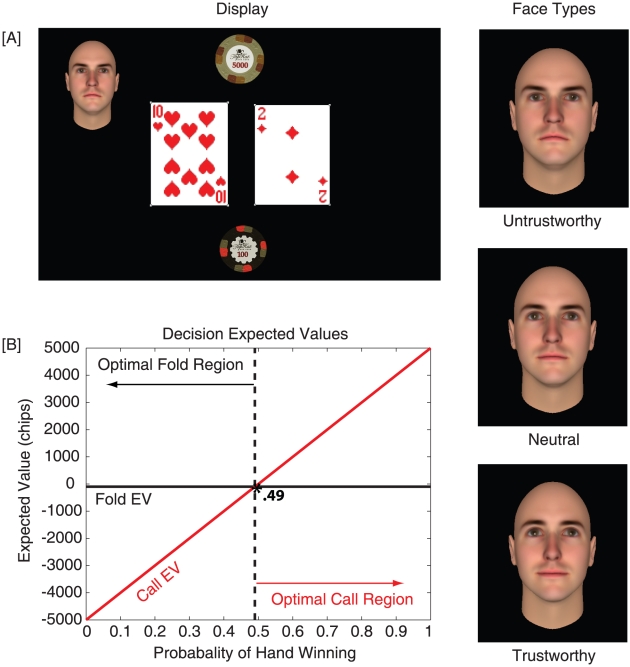
Diagram of the display viewed by participants in this experiment, and expected values for each of the two possible decisions. [A] Participants played a simplified version of Texas Hold'em poker and were provided information about their starting hand and the opponent who was betting. Based on this information, they were required to make call/fold decisions. If participants choose to fold, they are guaranteed to lose their blind (−100 chips), whereas if they choose to call, they have a chance to either win or lose the bet amount (5000 chips) that is based on the probability of their hand winning against a random opponent. Opponent faces were obtained from an online database [26], [27]. The right column of the figure shows one face identity for three different trustworthiness values. [B] Graph shows how the expected value for each decision changes across starting hands. The ‘optimal decision’ would be the one that results in the greatest expected value. Therefore, participants should fold when the probability of their hand winning is below .49, and call if it is greater. See the Experimental Methods Section for additional details.

To investigate if people are inferring their opponent's style through face information, participants competed in a simplified poker game (Figure 2) against opponents whose faces varied along an axis of trustworthiness [26], [27]: untrustworthy, neutral, and trustworthy. If people use information about an opponent's face, it predicts they should *systematically* adjust their wagering decisions, despite the fact that they receive no feedback about outcomes, and the value associated with the gambles is identical between conditions. Conversely, if people only use outcome-based information in competitive games, or use face information inconsistently, then there should be no reliable differences in wagering decisions between the groups.

## Methods

### Participants

14 adults signed written consent to participate in this study for monetary compensation (see Procedure). Participants were between 19 and 36 years of age, and all had normal or corrected-to-normal vision. In order to be eligible for this study, participants achieved a minimum score of 7/10 on a pre-experimental exam about the rules of Texas Hold'em poker (See Materials S1), in addition to demonstrating no previous history of gambling addiction. The experimental protocol used in this study passed a written Harvard University Human Subjects Review Committee.

Data from a pre-experimental inventory found that participants were novice poker players as 12 of the 14 in this study played less than 10 hours/year. Moreover, all participants in this study tended to play more ‘live’ games than online games. In fact, 12 of 14 participants played more than 90% of their games ‘live’, rather than online.

### Materials and Apparatus

Participants saw a simple Texas Hold'em scenario that was developed using MATLAB's psychtoolbox, running on a Mac OSX system. The stimuli consisted of the participant's starting hand, the blind and bet amounts, in addition to the opponent's face (Figure 2A). Note that this set-up strips-away or controls for much of the information that is commonly used by poker players when making a decision, which is outside the focus of our experimental question (e.g., position in the sequence of betting, size of the chip stack, the number of active players in the pot, etc.).

### Face trustworthiness

The opponent's faces were derived from an online database that morphed neutral faces along an axis that optimally predicts people's subjective ratings of trustworthiness [27]. More specifically, faces in the trustworthy condition are 3 standard deviations above the mean/neutral face along an axis of trustworthiness. Whereas, untrustworthy faces are 3 standard deviations below the mean/neutral face along this dimension.

The database provided 100 different ‘identities’. Each of the faces was morphed to three trust levels, giving a neutral, trustworthy and untrustworthy exemplar for each face. Therefore, in this experiment, there were 300 total trials (100 identities ×3 trust levels each), that were presented in a random order.

### Hand distribution

Two-card hand distributions were selected to be identical between levels of trustworthiness. In order to minimize the probability that participants would detect this manipulation, we used hand distributions that had identical value, but are different in their appearance (e.g., cards were changed in their absolute suit (i.e., hearts, diamonds, clubs, spades) without changing the fact that they were suited (e.g., heart, heart) or unsuited (e.g., heart, club). This precaution seemed to work as no participant reported noticing this manipulation.

Within each level of trustworthiness, we also selected hand distributions to have an equal number of optimal call (i.e., accepting a bet) and fold (not accepting a bet) decisions (50 call/50 fold). Optimal decisions are considered to be the decision that maximizes the expected value (i.e., the number of chips earned; See Figure 2B). The expected value associated with folding is always negative 100 chips since it results in the guaranteed loss of the initial bet (or ‘blind’), which is 100 chips, regardless of the starting hand. Conversely, when calling the opponent's bet (100 blind +4900 call amount = 5000 chips), the expected value is based on the probability of the hand winning. Since opponents in this experiment are random, the probability of the hand winning was determined by simulating the player's starting hand against every possible random opponent's hand and community card combination. Therefore, optimal call decisions are those that the expected value for calling exceeds negative 100 chips (i.e., the expected value for folding). Against a random opponent, Figure 2B shows that optimal play would require people to call with hand winning probabilities that exceed .49, and fold otherwise. The bet size of 5000 chips was an attempt to maximize the number of possible hands in each of the optimal decision regions.

### Procedure

After participants passed the Texas Hold'em exam and signed the consent form, they were provided task with instructions. The instructions explained that they would be participating in a simplified version of Texas Hold'em poker. Unlike ‘real’ poker, they would always be in the big blind (i.e., they were required by the rules to make an initial bet of 100 chips) facing only one opponent who always bets 5000 chips.

Moreover, they would only be allowed two possible decisions: call or fold. Therefore, unlike ‘real’ poker, they would not be able to ‘bluff’ their opponent out-of the pot or ‘out-play’ their opponent since no extra cards are dealt. Participants were instructed that the only information they available have to make their betting decisions is their starting hand and the opponent who is betting. It was explained that similar to ‘real’ poker, different opponents may have different ‘styles’ of play. We did *not* mention anything about the opponent's face or the trustworthiness of the opponent. They were only told that if they choose to call, the probability of their hand winning is going to be based on their starting hand and their opponent's style. Of course, unknown to them, the opponents were always betting randomly in this study.

Unlike ‘real’ poker, no feedback about outcomes was provided after each trail and no ‘community cards’ were dealt. Rather, the hand was simulated and the outcome was recorded to use for consideration in their bonus pay. Participants received bonus pay that is based on the outcome of one randomly selected trial from the 300 possible hands. If participants chose to call the randomly selected trial, and the outcome was a win, they would earn a total of $15 ($5 participation + $5 gambling allowance + $5 bonus). Whereas, if participants decided to call and the outcome is a loss, then they would only earn $5 ($5 participation + $5 gambling allowance - $5 bonus). Finally, if participants chose to fold the randomly selected hand, they would earn $10 ($5 participation + $5 gambling allowance + $0 bonus: –$.10 which participants understood would be rounded to nearest dollar amount). Therefore, participants were motivated to make optimal decisions, as that would maximize their chance of winning bonus money. After completing the 300 trials, participants were paid and debriefed.

## Results

### Reaction time

**Figure 3:**
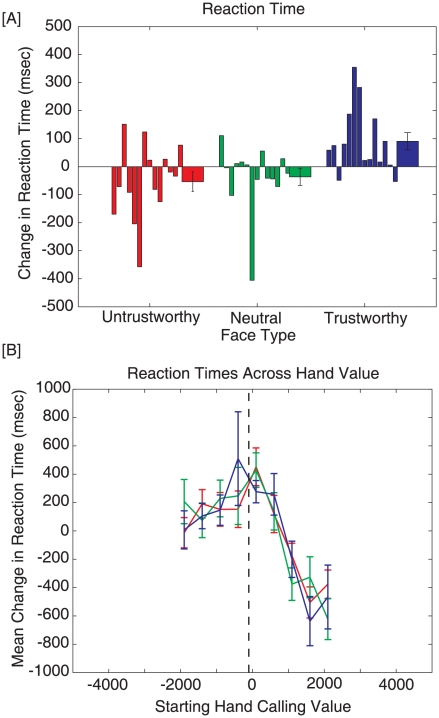
Figure demonstrates changes in reaction times. For Panel A, the first 14 bars reflect individual participant data, while the last bar represents the average for each condition (Error bars represent ± SEM). [A] Change in reaction time across face conditions. Participants took significantly longer to make a decision against a trustworthy opponent (Blue) than untrustworthy (Red) and Neutral (Green) opponents. [B] Mean change in reaction time across starting hand value. People took significantly longer (collapsing across trustworthiness conditions) to make decisions for hands in the optimal fold region (left of black dashed line) than hands in the optimal call region (right of dashed line). Moreover, differences between trustworthiness groups were most pronounced around the decision boundary.

Figure 3 shows average changes in reaction time across face conditions ([A]) and hand value ([B]). Reaction time is defined to be the interval between display onset and the time of decision. Change in reaction time is computed for each participant by calculating the mean reaction time in each face condition and subtracting-off the overall mean reaction time, across conditions. These means are then averaged across participants, to produce the graph in Figure 3A. This procedure simply adjusts for the differences in baseline reaction time across different participants (i.e., transform to zero mean) and allows us to assess the impact of face-type on changes in reaction time, independent of differences in absolute levels of reaction time. A similar procedure of normalizing behavioral data within a subject, to permit comparison across the trust levels and hand qualities, was followed whenever ‘changes’ in a dependent measure are discussed.

Figure 3A shows that participants took longer to react to trustworthy opponents (Blue; Mean = 38.08 msec, SE = 23.94 msec) than to neutral opponents (Green; Mean = −35.76 msec, SE = 25.24 msec) and untrustworthy opponents (Red; Mean = −48.48 msec, SE = 24.74 msec). A Friedman's test found a significant main effect of trustworthiness on reaction time, χ^2^(2)  = 7.00, p = .03. Note that Friedman's test is used throughout this paper due to violations of the normality assumption in our data that is required by a repeated measures ANOVA. A Wilcoxon signed-rank test was used as a post-hoc test and demonstrated that the reaction times against a trustworthy opponent are significantly more than untrustworthy (p = .03) and neutral (p = .03) opponents. No other pairwise differences were found.

Figure 3B demonstrates changes in reaction time against starting hand value. It is clear that reaction times across hand value are relatively consistent across face-types. However, any differences in reaction time across levels of trustworthiness tend to occur near the optimal decision boundary (Black dashed line). Moreover, it is evident that people are taking longer (collapsing across trustworthiness levels) on average to react to hands in the optimal fold region (4 lowest bins; Mean = +167.47 msec, SE = 44.90) than to hands in the optimal call region (5 highest bins; Mean = −133.97 msec, SE = 42.33). A Wilcoxon signed-rank test proved this difference to be significant (p<.01).

### Correct decisions

**Figure 4:**
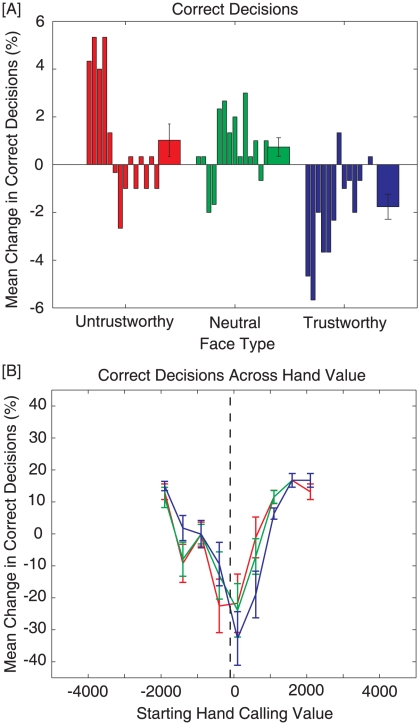
Figure shows changes in correct decisions. For Panel A, the first 14 bars reflect individual participant data, while the last bar represents the average for each condition (Error bars represent ± SEM). [A] Change in correct decisions across face types. Participants made significantly more mistakes against trustworthy opponents (Blue) than neutral (Green) and untrustworthy (Red) opponents. [B] Mean change in correct decisions across starting-hand value. People did significantly worse (collapsing across trustworthiness conditions) for hands near the optimal decision boundary. Differences between groups were also more pronounced for these mid-value hands.

Figure 4 displays mean change in percent correct decisions across levels of trustworthiness ([A]) and hand value ([B]). A correct decision was defined to be the decision that results in the greatest expected value (Figure 4B). Figure 3A shows that participants made significantly more mistakes against trustworthy opponents (Blue; Mean = −1.76%, SE = .55%) than neutral (Green; Mean = .74%, SE = .40%) and untrustworthy opponents (Red; Mean = 1.02%, SE = .70%). A Friedman's test found a significant main effect of trustworthiness on correct decisions, χ^2^(2)  = 8.32, p = .02. Wilcoxon signed-rank test was used as a post-hoc test demonstrated that participants made significantly more mistakes against trustworthy opponents than neutral (p<.01) and untrustworthy (p = .04) opponents. No other pairwise differences were observed.

Figure 4B depicts changes in correct decisions across hand value. The effects of face-type on correct decisions seem to be the most pronounced near the optimal decision boundary. Moreover, people tended to make the most mistakes (collapsing across trustworthiness levels) for medium-valued hands (middle 3 bins; Mean = −16.71%, SE = 2.59%) over low-value (lowest 3 bins; Mean = 2.66%, SE = 1.47%) and high-valued (highest 3 bins; Mean = 14.05%, SE = .75%) hands. A Friedman's test found a significant main effect of hand value on correct decisions, χ^2^(2)  = 91.07, p<01, and a Wilcoxon signed-rank test showed that all of the pairwise differences were significant (p<.01).

### Betting behavior

**Figure 5:**
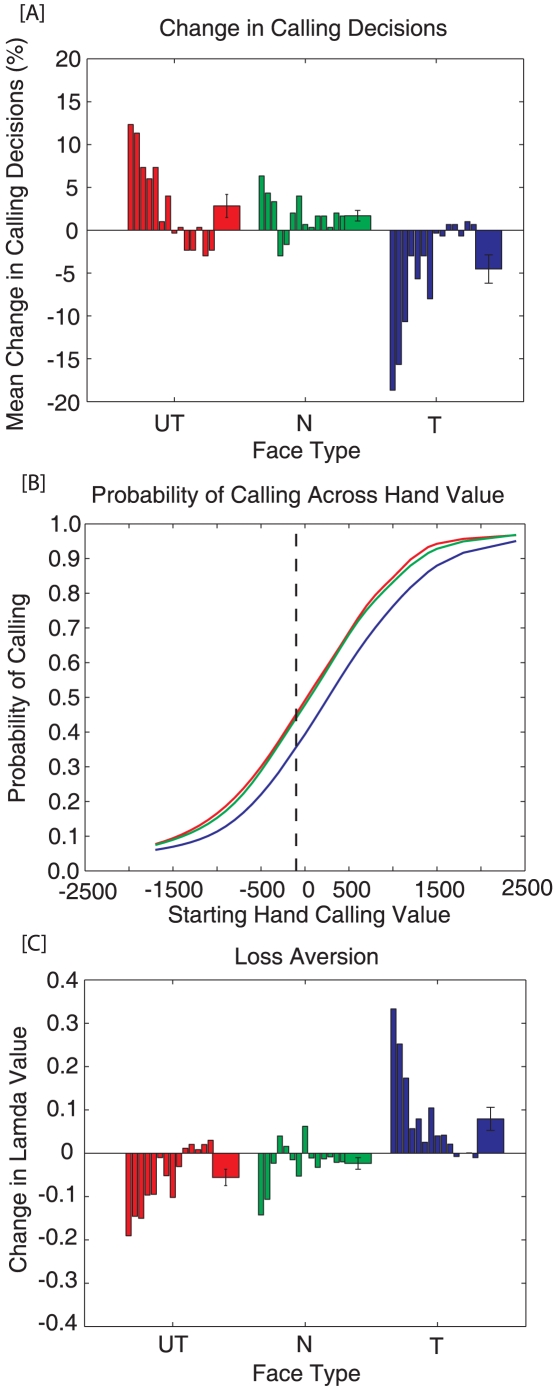
Changes in calling behavior and loss aversion parameters across faces types. In Panel A, the first 14 bars reflect individual participant data, while the last bar represents the average for each condition (Error bars represent ± SEM). [A] Change in calling decisions across face types. Participants called significantly less against trustworthy opponents (Blue) than neutral (Green) opponents. [B] The observed changes in calling resulted from a shift in the average calling function for trustworthy faces. This suggests that participants ded a staneerting hand with greater expected value in order to call at similar rates against a trustworthy opponent. [C] Change in lambda values for the utility fits across face conditions. The results show that lambda values are significantly greater against trustworthy opponents than against neutral or untrustworthy opponents. Moreover, subjects are gain-loss neutral, unless they are playing a trustworthy opponent, when they show significant loss aversion.

Figure 5 shows mean changes in percent calling behavior ([A,B]) and differences in loss aversion parameters ([C]) across trustworthiness levels. Please note that, in poker, accepting an opponent's bet is termed *calling*, while not accepting their bet is termed *folding*. Figure 5A demonstrates that people call less against trustworthy opponents (Blue; Mean = −4.52%, SE = 1.72%) than against neutral (Green; Mean = 1.69%, SE = .64%) and untrustworthy opponents (Red; Mean = 2.83%, SE = 1.40%). A Friedman's test found a significant main effect of trustworthiness on calling behavior, χ^2^(2)  = 10.51, p = .01. A Wilcoxon signed-rank test demonstrated that participants folded significantly more against trustworthy opponents than neutral (p<.01) opponents, but not untrustworthy (p = .06) opponents, although a trend was observed. No other pairwise differences were found.

In order to directly investigate how opponent information is impacting wagering decisions, a softmax expected utility model (Materials S1; Figure S2) was used that separates the influence of three different choice parameters: a loss aversion parameter (lambda), a risk aversion parameter (rho), and a sensitivity parameter (gamma). These parameters have been shown to partially explain risky choices with numerical outcomes in many experimental studies, and in some field studies [28]. They were fit to each subject's data and averaged across subjects to explore the impact of opponent information on components of risk and loss preference revealed by wagering.

Figure 5B shows the average probability of calling across the three different opponent conditions. The curves show that participants required a higher-value hand to call (at similar levels) against a trustworthy opponent (Blue Curve) than a neutral (Green Curve) or untrustworthy (Red Curve) opponent. For example, at a 50% calling rate, it requires a hand with an expected value of 0 chips against a neutral and untrustworthy opponent, and a hand with an expected value of positive 300 chips against a trustworthy opponent.

The loss aversion parameter discussed above provides a way to directly quantify this ‘shift’ in calling decisions. Figure 5C depicts the average lambda values across levels of trustworthiness. It was found that participants showed greater loss aversion against trustworthy opponents (Blue; Mean = .08, SE = .03) than against neutral (Green; Mean = −.02, SE = .01) and untrustworthy opponents (Red; Mean = −.05, SE = .02). A Friedman's test found a significant main effect of trustworthiness on lambda values, χ^2^(2)  = 9.00, p = .01. A Wilcoxon signed-ranks test demonstrated that people showed significantly more loss aversion against trustworthy opponents than neutral (p = .01) and untrustworthy (p = .02) opponents. Moreover, If people are weighting gains greater than losses (gain seeking) lambda values should be significantly less than one, whereas if people weight gains and losses equally, the lambda value should be statistically equal to one (gain-loss neutral). However, if people are trying to avoid losses, the lambda value should be significantly above one (loss aversion). Lambda values were significantly above 1 (gain-loss neutral point) against trustworthy opponents (Mean = 1.14, SE = .06, p = .04), but not against neutral (Mean = 1.04, SE = .06, p = .55) or untrustworthy opponents (Mean = 1.00, SE = .06, p = .96). This suggests that people show significant loss aversion when playing trustworthy opponents, but not against neutral or untrustworthy opponents. If the loss aversion for each subject is corrected, differences in mistakes between conditions disappears. No significant differences were found across trustworthiness conditions for risk aversion (rho), χ^2^(2)  = 3.57, p = .17, or sensitivity (gamma), χ^2^(2)  = 3.00, p = .22.

## Discussion

From these results, it is clear that people are using face information to modify their wagering decisions in a competitive task. These results can be easily framed within a Bayesian interpretation [29]–[31] and are related to ideas in Bayesian explaining away. Since an opponent's ‘style’ is a hidden state, participants must estimate it through observable variables. For example, a Bayesian estimator could assume that an opponent is random (i.e., they bet uniformly across hand value) until information to the contrary is acquired. In our experiment, the only information participants have available about their opponent's style is the trustworthiness expressed by their face. If people are using beliefs that trustworthy opponents tend to bet with high-value hands, then they should fold more frequently than against a random opponent. Indeed, participants' observed changes in betting behavior (Figure 5) are in agreement with this interpretation. If feedback about outcomes or information about an opponent's hand (e.g., during a showdown) were available, a Bayesian estimator would use this information to update its beliefs about the opponent, forming a posterior estimate to use for the next hand. This predicts that face information should carry greater weight for betting behavior when there is little or no additional data about an opponent available (e.g., our experiment) or with extremely noisy opponent data (e.g., novice who doesn't know how to interpret this information). It is also worth noting that even though the relative increase in errors (∼3%) against trustworthy opponents seems small (Figure 4A), the average return on investment for the most elite online poker players is only 6.8% [32]. Therefore, an increase in mistakes of this magnitude could lead to significant decreases in a player's earnings over time.

Another interpretation of this data is that people are acting irrationally by becoming more loss averse against trustworthy appearing opponents. This possibility is evidenced by increases in people's loss aversion parameters as estimated by the softmax utility model (Figure 5B,C). Although distinguishing between the rational (Bayesian) interpretation and the irrational (utility) interpretation is an important and interesting question, this experiment is unable to discriminate between the two alternatives. It is apparent that people are adjusting their wagering behavior against opponents whose faces correlate with trustworthiness, although the reason for this change in behavior is unclear. Future studies will more directly explore this distinction.

Although the faces used in this experiment are thought to optimally predict subjective ratings of trustworthiness, it is also known that impressions of trust are deeply related to other attributes, such as perceived happiness, dominance, competence, etc. [27]. To investigate the possible role of these attributes, we conducted an independent rating task (Materials S1; Figure S1) using a different group of subjects and correlated these results with the wagering behavior observed in this study (Materials S1). The results demonstrate that the impressions of trustworthiness also influence impressions of many other attributes that correlate with wagering decisions. Therefore, a more general conclusion is that common avoidance cues (dominant, angry, masculine) lead to more aggressive wagering decisions (i.e., increased calling), whereas approach cues (happy, friendly, trustworthy, attractive) tend to lead to conservative wagering decisions (i.e., increased folding). Although this seems contrary to evolutionary predictions, it is rational within the context of poker since approach cues may suggest the opponent has a good hand and/or is less likely to bluff. This interpretation is supported by the fact that subjects were more likely to call against opponents who were perceived to frequently bluff, and these opponents have similar subjective impression rating trends as those who are high on avoidance dimensions (See Figure S1).

The increased influence of trustworthiness on reaction time (Figure 3B) and correct decisions (Figure 4B) around the optimal decision boundary suggests that people are using face information most for medium-value hands. This could be explained by optimal data fusion [33]–[35], which states that the more uncertainty people have about the value of their hand, the more they should weigh face information when making a betting decision. Since participants in our experiment were novices (12 of 14 play less than 10 hours/year), they may have a more reliable estimate of high-value hands since those tend to be more salient/memorable (e.g., face cards, aces, pairs, etc.) than medium- and low-value hands. Indeed, participants in our study took significantly longer to react to hands in the optimal fold region (Figure 3B), and also made significantly more mistakes for medium- and low-value hands (Figure 4B), supporting this notion.

It is also interesting that all of the changes in wagering decisions were observed against trustworthy opponents, while untrustworthy opponents did not yield any significant results. This asymmetry is even more fascinating given that people's perception of trustworthiness is more sensitive to changes between untrustworthy and neutral faces, than between neutral and trustworthy faces [27]. One possible explanation stems from the assumption that people use a random opponent decision criterion in this task, unless there is information that an opponent is betting with non-random hands. In this respect, neutral and untrustworthy faces are functionally the same: neutral faces do not provide information about an opponent's style, while untrustworthy faces may suggest that opponents are betting with poor hands. However, if participants are already assuming opponents bet randomly, they cannot decrease their criterion any further. In agreement with this proposal, Figure 5B shows that the inflection point for the neutral (Green) and untrustworthy (Red) curves is very close to the optimal decision boundary for a random opponent. However, trustworthy faces may provide information that the opponent has a high-value hand, leading to the observed shift towards more conservative wagering behavior.

Another direction of future research will investigate if changes in people's wagering decisions against trustworthy opponents resulted from an explicit strategy, or an implicit reaction to the faces. Although we have been interpreting the results with respect to normative decision theory, research has also demonstrated that impressions of trust can occur extremely rapidly [5]–[8], [36], and that implicit information can also modify brain activity and behavior [37]–[40]. In fact, research has also shown that loss aversion is tightly related to emotional arousal [28], suggesting the loss aversion observed against trustworthy opponents (Figure 5C) could be an implicit reaction. Therefore, future research will explore if these changes in wagering decisions are a conscious strategy or an automatic response.

In conclusion, we have shown that rapid impressions of opponents modify wagering decisions in a zero-sum game with hidden (opponent) information. Interestingly, contrary to the popular belief that the optimal poker face is neutral in appearance, the face that invokes the most betting mistakes by our subjects is has attributes that are correlated with trustworthiness. This suggests that poker players who bluff frequently may actually benefit from appearing trustworthy, since the natural tendency seems to be inferring that a trustworthy-looking player bluffs less. More generally, these results are important for competitive situations in which opponents have little or no experience with one another, such as the early stages of a game, or in one-shot negotiation situations among strangers where ‘first impressions’ matter.

## Supporting Information

Materials S1 Supporting results from additional study and details about the utility model.
(0.08 MB DOC)

Figure S1 Depicts the difference in mean rating value between the two most extreme values of face trustworthiness (trustworthy - untrustworthy) for each question, averaged across subjects. It is apparent from the figure that peoples' subjective impressions of happiness (Question 6), anger (Question 7), and friendliness (Question 8) were most influenced by changes in face-type between trustworthy to untrustworthy faces. (B) Demonstrates correlations between the mean changes in calling behavior across the three levels of trustworthiness, reported in Figure 5A, and the mean perceptual rating on each question, averaged across subjects. It is apparent that subjects were more likely to call when faces had common “avoidance” signals (red color items): masculine, dominant, and angry. Whereas, subjects were less likely to call when opponent's faces contained common “approach” signals (yellow color items): attractive, trustworthy, happy, and friendly. Moreover, people were more likely to call against opponents who were perceived as someone who frequently bluffs (green item).
(0.41 MB PDF)

Figure S2 Characteristics of the softmax utility model. (A) Individual calling behavior for each of the 14 participants, across all three trustworthiness conditions: untrustworthy (red curve), neutral (green curve), and trustworthy (blue curve). Calling behavior versus expected value tends to vary across subjects, although average performance demonstrates systematic differences against trustworthy opponents (Figure 4). (B) Scatter plot of risk aversion (rho) and loss aversion (lambda) fit values for each of the 14 participants, across all three conditions. Using all the data, there is a significant correlation between loss aversion and risk aversion fit values (r = .40, p<.01). However, if the smallest rho values (points within red dashed box) are removed from analysis, there is no significant correlation between the utility parameters (r = .22, p = .19). (C) Using these utility parameters leads to calling behavior that is much closer to optimal behavior. This suggests that loss and risk aversion accounts for the observed changes in calling behavior (Figure 4). (D) Average calling behavior across utility is much closer to optimal performance than compared to Figure 4. Moreover, there are fewer differences in calling between conditions.
(1.02 MB PDF)
