# Supplementary material for: Human Wagering Behavior Depends on Opponents' Faces
Source: PLoS One. 2010 Jul 21;5(7):e11663. doi: 10.1371/journal.pone.0011663 (PMC2908123; doi:10.1371/journal.pone.0011663)
Supplement: Materials S1 — Supporting results from additional study and details about the utility model. (0.08 MB DOC) [file pone.0011663.s001.doc]

**Materials S1**

**Texas Hold’em Evaluation**

**This inventory is meant to assess that you understand the basic rules and procedures involved with Texas Hold’em. We need to assure that prospective subjects have, at least, a basic-level of understanding before we can allow participation.**

**To participate in this experiment, you will need to score 7/10, or above.**

1) How many cards are dealt on the ‘flop’?

a. 1 b. 2 c. 3 d. 4 e. 5

2) How many cards are dealt on the ‘turn’?

a. 1 b. 2 c. 3 d. 4 e. 5

3) How many ‘hole cards’ do players get at the beginning of each round?

a. 1 b. 2 c. 3 d. 4 e. 5

4) If the blind structure is $5/$10, what amount does the ‘big blind’ need to contribute?

a. $5 b. $10 c. $15 d. $20 e. Ante

5) Which of the following hands is most powerful (i.e., would win)?

a. pair b. 2 pair c. full house d. 3 of a kind e. flush

6) Which of the following hands is most powerful (i.e., would win)?

a. pair b. straight c. 3 of a kind d. 2 pair e. high card

7) If you have no money in the pot, and your opponent raises you to $5000, what is the

minimum amount you need to contribute to continue playing the hand?

a. $1000 b. $5000 c. $10,000 d. $15,000 e. $20,000

8) What is the number of cards you can use to make your ‘best hand’?

a. 3 b. 4 c. 5 d. 6 e. 7

9) What is the total number of community cards dealt-out in a round (flop, turn, river)?

a. 3 b. 4 c. 5 d. 6 e. 7

10) Who is your favorite poker player? (+1 regardless of answer)

**Please answer each of the following questions for every face you see by recording your answer on the response sheet.**

1) How masculine or feminine does this person look?

1 2 3 4 5 6 7

Very Average Very

Feminine Masculine

2) How attractive does this person look?

1 2 3 4 5 6 7

Very Average Very

Unattractive Attractive

3) How dominant or submissive does this person look?

1 2 3 4 5 6 7

Very Average Very

Submissive Dominant

4) How competent does this person look?

1 2 3 4 5 6 7

Not Very Average Very

Competent Competent

5) How trustworthy does this person look?

1 2 3 4 5 6 7

Very Average Very

Untrustworthy Trustworthy

6) How happy does this person look?

1 2 3 4 5 6 7

Not Very Average Very

Happy Happy

7) How angry does this person look?

1 2 3 4 5 6 7

Not Very Average Very

Angry Angry

8) How friendly does this person look?

1 2 3 4 5 6 7

Not Very Average Very

Friendly Friendly

9) Is this person likely to bluff* in a poker game?

1 2 3 4 5 6 7

Not Very Average Very

Likely Likely

* In card games, bluffing is an attempt to deceive your opponent by betting as if you have a strong hand when, in fact, you have cards that have a low-probability of winning.

*Experimental Face Rating Task*

**Subjects:** 15 subjects from Caltech voluntarily participated in the rating task after signing an informed consent form that was approved by California Institute of Technology’s Human Subjects Review Board.

**Methods and Procedure:** All of the subjects viewed a group of 30 faces that were a subset of those used in the poker experiment (10 from each of the three levels of trustworthiness). Subjects were asked to rate each of the 30 faces on nine different attributes (See Appendix B), and were allowed as much time as needed to make their ratings. Subjects’ ratings were recorded and averaged for each of the three different levels of trustworthiness. The result was three mean rating scores for each subject and question. These mean scores were used to produce Figure S1.

**Results:**

**Figure S1. [A]** Depicts the difference in mean rating value between the two most extreme values of face trustworthiness (trustworthy – untrustworthy) for each question, averaged across subjects. It is apparent from the figure that peoples’ subjective impressions of happiness (Question 6), anger (Question 7) and friendliness (Question 8), were most influenced by changes in face-type between trustworthy to untrustworthy faces. **[B]** Demonstrates correlations between the mean changes in calling behavior across the three levels of trustworthiness, reported in Figure 5A, and the mean perceptual rating on each question, averaged across subjects. It is apparent that subjects were more likely to call when faces had common ‘avoidance’ signals (red color items): masculine; dominant; and angry. Whereas, subjects were less likely to call when opponent’s faces contained common ‘approach’ signals (yellow color items): attractive; trustworthy; happy and friendly. Moreover, people were more likely to call against opponents who were perceived as someone who frequently bluffs (green item).

**Conclusion:** Although the faces used in this experiment have been shown to optimally predict peoples’ subjective impressions of trustworthiness (Oosterhof & Todorov, 2008), it is apparent that impressions of other attributes are also strongly influenced by this manipulation (Figure S1). Moreover, it is evident that wagering behavior strongly correlates with many of these factors. Therefore, a more general conclusion is that common avoidance cues seem to lead to more aggressive wagering decisions (i.e., increased calling), whereas approach cues tend to lead to conservative wagering decisions (i.e., increased folding). Although this seems contrary to evolutionary predictions, it is rational within the context of poker since approach cues may suggest the opponent has a better hand and/or is less likely to bluff. This interpretation is supported by the fact that subjects were more likely to call against opponents who were perceived as someone who frequently bluffs (green correlation).

*Softmax Utility Model*

Participants in this study were choosing between a stochastic gain/loss (calling) and a sure loss (folding). In order to explore how levels of trustworthiness influenced wagering decisions, a utility model was used that is able to determine three aspects of choice behavior (35): a) Loss aversion (lambda) - relative weighting between losses (L) and gains (W); b) Risk aversion (rho) – changes in choice sensitivity due to changes in absolute value; and c) Sensitivity (gamma) – consistency in choices between the sure loss (C) and the stochastic gamble (Figure S2).

Participants’ utility functions are represented in a 2-part power function (19,35):

$U(x)=\left\{\frac{x(w)^{\rho}}{-\lambda|x(l)|^{ \rho}} \right.\frac{if x\geq0}{if x<0}$ **[1]**

In our experiment, the gain amount (x(w)) was always +5000 chips, and the loss amount (x(l)) was always -5000 chips. In order to determine the probability that participants will accept a stochastic gamble (c - call) over a sure loss (f - fold), we used a logit softmax function:

$\prod_{i}^{N} p_{i}(c>f)^{nc}p_{i}(c<f)^{nf}$ **[2]**

where *nc* is an indicator variable reflecting how many times the participant called and *nf* is the number of times the participant folded across trials (*i*). p(c>f) is the probability that the subject called, and is given by:

$p(c>f)=\frac{1}{1+e^{\gamma}[U(x(c_{i}))-(p(x(w_{i}))*U(x(w_{i})))+((1-p(x(w_{i})))*U(x(l_{i}))]}$ **[3]**

where *x(c)* is the sure loss amount (-100 chips), *p(x(w_i_))* is the probability of the hand winning, and gamma is the logit sensitivity parameter.

The fit of each parameter was obtained using MATLAB’s unconstrained nonlinear optimization (fminsearch). Standard errors were achieved by bootstrapped estimates from each subjects data. Utility parameters were fit for choice data in each condition separately to assess the impact of trustworthiness on the utility parameters. Averages across subjects were then accomplished by the weighted sum of each parameter:

$\bar{x}=\sum_{j=1}^{N} \left(\frac{1}{\sigma_{\bar{x}}^{2}}\right)_{j}y_{j}$ **[4]**

where the weights are given by the inverse squared error (i.e., the reliability) associated with the utility parameter *y*, for the *j*th participant.

**Figure S2.** Characteristics of the softmax utility model. [A] Individual calling behavior for each of the 14 participants, across all three trustworthiness conditions: untrustworthy (red curve); neutral (green curve); and trustworthy (blue curve). Calling behavior versus expected value tends to vary across subjects, although average performance demonstrates systematic differences against trustworthy opponents (Figure 4). [B] Scatter plot of risk aversion (rho) and loss aversion (lambda) fit values for each of the 14 participants, across all three conditions. Using all the data, there is a significant correlation between loss aversion and risk aversion fit values (r=.40, p<.01). However, if the smallest rho values (points within red dashed box) are removed from analysis, there is no significant correlation between the utility parameters (r=.22, p=.19). [C] Using these utility parameters leads to calling behavior that is much closer to optimal behavior. This suggests that loss and risk aversion accounts for the observed changes in calling behavior (Figure 4). [D] Average calling behavior across utility is much closer to optimal performance than compared to Figure 4. Moreover, there are fewer differences in calling between conditions.
