# Supplementary figures and images for: Human Wagering Behavior Depends on Opponents' Faces
Source: PLoS One. 2010 Jul 21;5(7):e11663. doi: 10.1371/journal.pone.0011663 (PMC2908123; doi:10.1371/journal.pone.0011663)

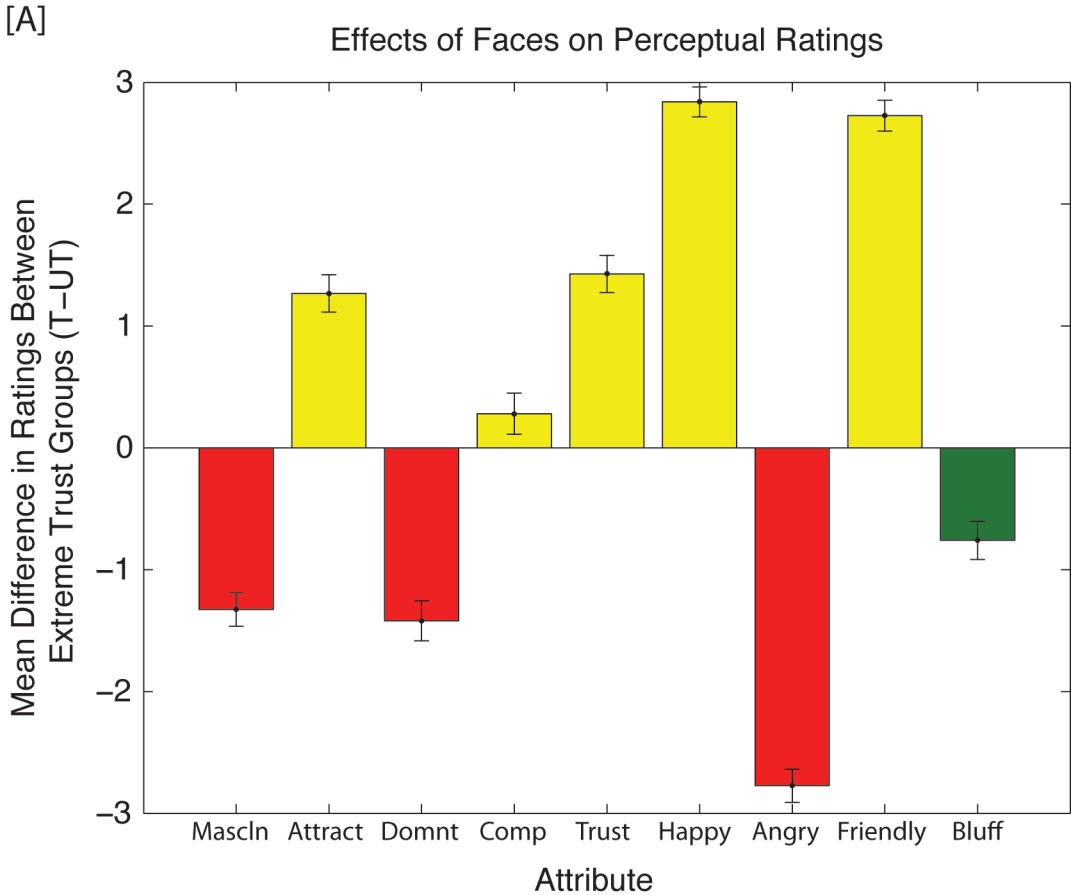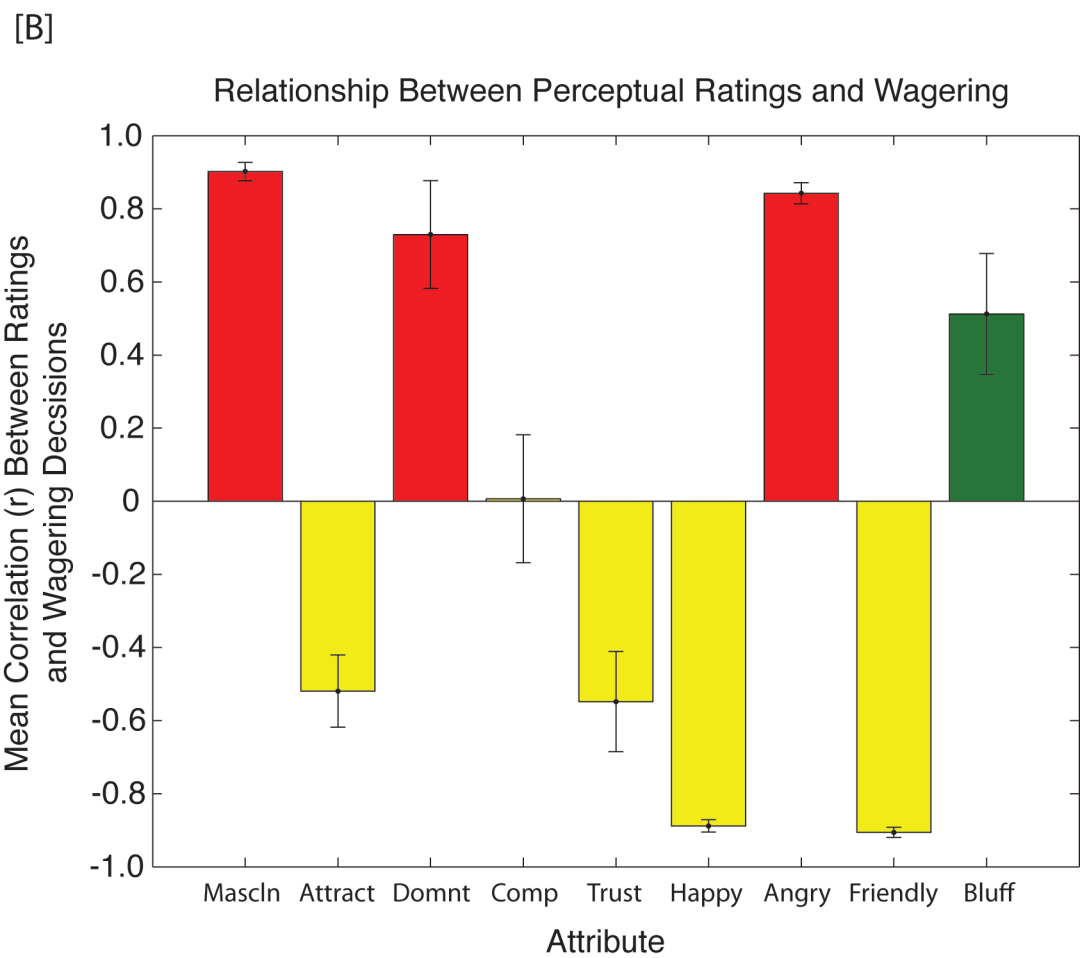

Supplement: Figure S1 — Depicts the difference in mean rating value between the two most extreme values of face trustworthiness (trustworthy - untrustworthy) for each question, averaged across subjects. It is apparent from the figure that peoples' subjective impressions of happiness (Question 6), anger (Question 7), and friendliness (Question 8) were most influenced by changes in face-type between trustworthy to untrustworthy faces. (B) Demonstrates correlations between the mean changes in calling behavior across the three levels of trustworthiness, reported in Figure 5A, and the mean perceptual rating on each question, averaged across subjects. It is apparent that subjects were more likely to call when faces had common “avoidance” signals (red color items): masculine, dominant, and angry. Whereas, subjects were less likely to call when opponent's faces contained common “approach” signals (yellow color items): attractive, trustworthy, happy, and friendly. Moreover, people were more likely to call against opponents who were perceived as someone who frequently bluffs (green item). (0.41 MB PDF) [file pone.0011663.s002.pdf]

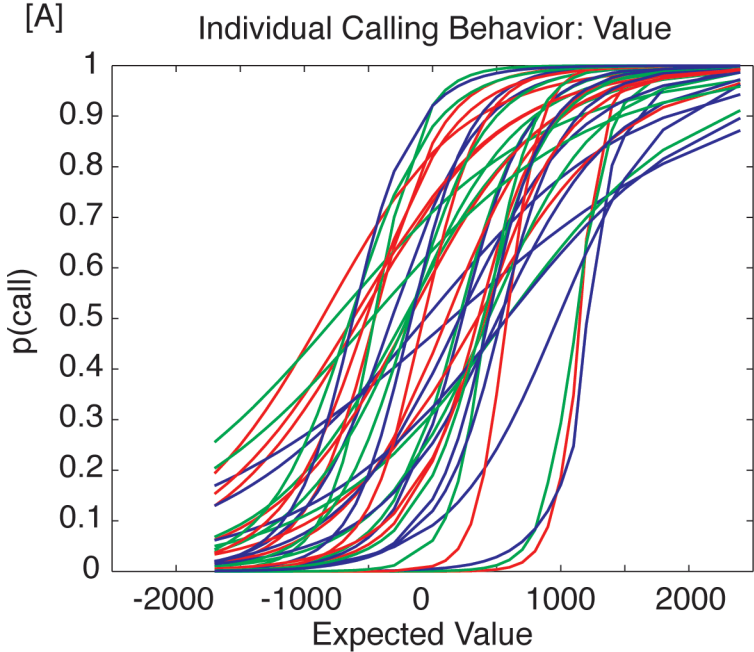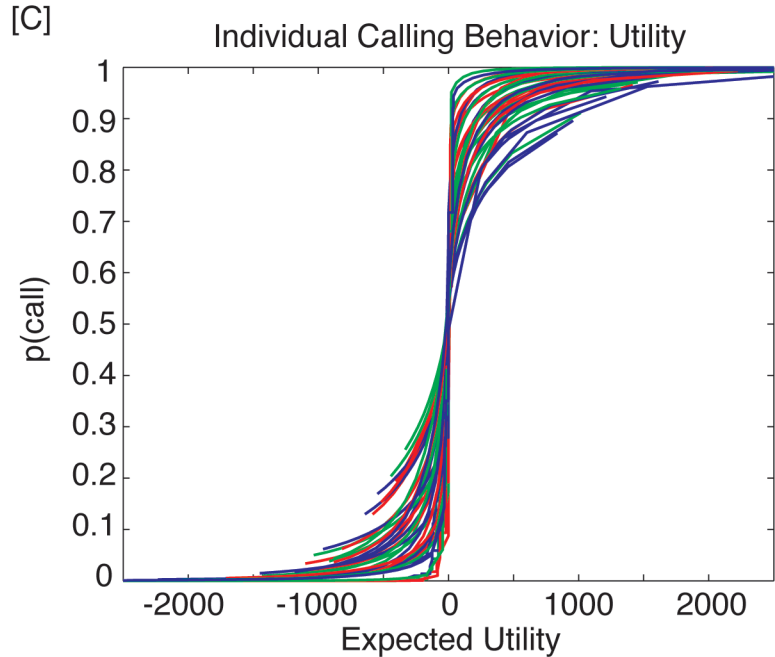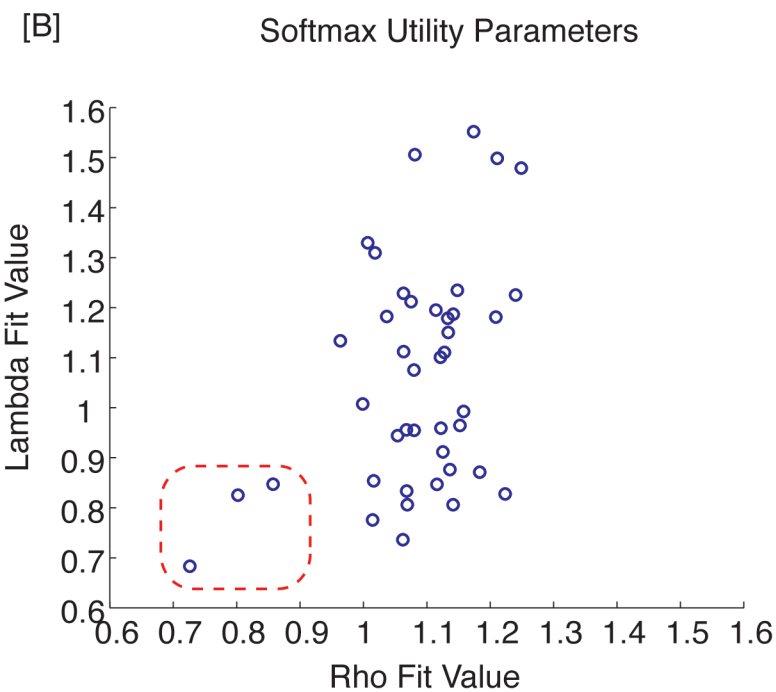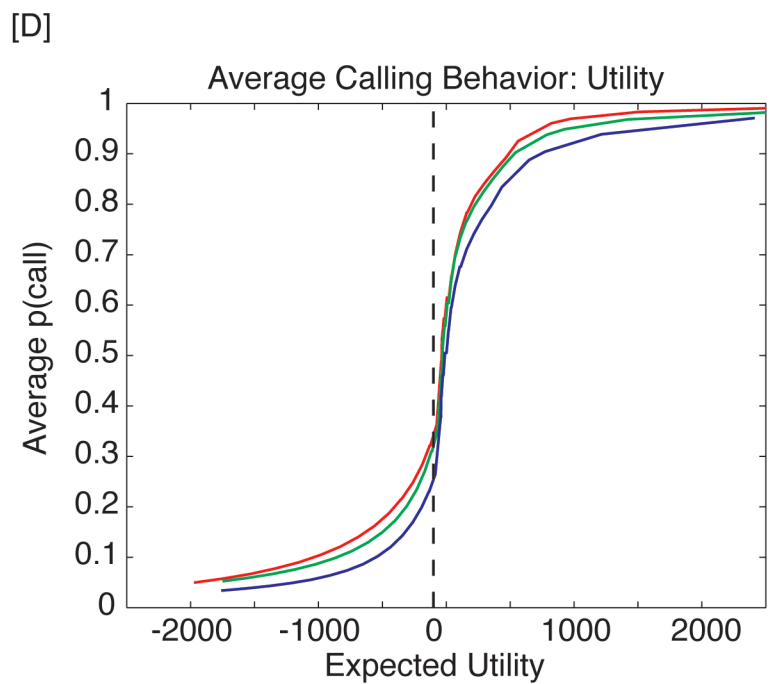

Supplement: Figure S2 — Characteristics of the softmax utility model. (A) Individual calling behavior for each of the 14 participants, across all three trustworthiness conditions: untrustworthy (red curve), neutral (green curve), and trustworthy (blue curve). Calling behavior versus expected value tends to vary across subjects, although average performance demonstrates systematic differences against trustworthy opponents (Figure 4). (B) Scatter plot of risk aversion (rho) and loss aversion (lambda) fit values for each of the 14 participants, across all three conditions. Using all the data, there is a significant correlation between loss aversion and risk aversion fit values (r = .40, p<.01). However, if the smallest rho values (points within red dashed box) are removed from analysis, there is no significant correlation between the utility parameters (r = .22, p = .19). (C) Using these utility parameters leads to calling behavior that is much closer to optimal behavior. This suggests that loss and risk aversion accounts for the observed changes in calling behavior (Figure 4). (D) Average calling behavior across utility is much closer to optimal performance than compared to Figure 4. Moreover, there are fewer differences in calling between conditions. (1.02 MB PDF) [file pone.0011663.s003.pdf]
